# Cell type- and density-dependent effect of 1 T static magnetic field on cell proliferation

**DOI:** 10.18632/oncotarget.14480

**Published:** 2017-01-04

**Authors:** Lei Zhang, Xinmiao Ji, Xingxing Yang, Xin Zhang

**Affiliations:** ^1^ High Magnetic Field Laboratory, Chinese Academy of Sciences, Hefei, Anhui, 230031, P. R. China; ^2^ University of Science and Technology of China, Hefei, Anhui, 230036, P. R. China

**Keywords:** static magnetic field, cancer cell, EGFR

## Abstract

Increasing evidence shows that static magnetic fields (SMFs) can affect cell proliferation but mixed results have been reported. Here we systematically examined the effects of 1 T (Tesla) SMF, which is close to the SMF intensity that patients are exposed to MRI (magnetic resonance imaging) scanners in hospitals, for its effect on 15 different cell lines, including 12 human and 3 rodent cell lines. Our results show that 1 T SMF does not have apparent impact on cell cycle or cell death. However, at higher cell density, it reduced cell numbers in six out of seven solid human cancer cell lines. We found that both cell type and cell density had evident impacts on SMF effects. Moreover, the EGFR-Akt-mTOR pathway, which varies significantly between different cell types and densities, contributes to the differential effects of SMF. In addition, SMF also increases the efficacy of Akt inhibitors on cancer cell growth inhibition. Therefore 1 T SMF affects cell proliferation in a cell type- and cell density-dependent manner, and the inhibition effect of 1 T SMF on multiple cancer cells at higher cell density may indicate its clinical potential in late stage cancer therapy.

## INTRODUCTION

Although there are numerous reports of *in vitro* and *in vivo* experiments that demonstrate the effects of magnetic field on biological systems, experimental coherence among different studies is still lacking. However, the seemingly inconsistent observations are mostly due to the different magnetic field parameters and multiple experimental variables. It is obvious that magnetic fields of different types (static or time-varying magnetic fields), field intensity (weak, moderate or strong magnetic fields) or frequencies (extremely low frequency, low frequency or radiofrequency) can lead to diverse and sometimes completely opposite results [1–4].

Besides various parameters of the magnetic fields, different biological samples in individual studies often have distinct genetic background, which makes them respond to the magnetic fields differentially. For example, Aldinucci et al. found that 4.75 T SMF significantly inhibited Jurkat leukemia cell proliferation but did not affect normal lymphocytes [5]. Rayman et al showed that growth of a few cancer cell lines can be inhibited by 7 T SMF [6], but other studies found that even 8-10 T strong SMFs did not induce obvious changes in non-cancer cells such as CHO (chinese hamster ovary) or human fibroblast cells [7, 8]. These results indicate that cell type is a very important factor that contributes to the differential cellular responses to SMFs. However, most individual studies investigated only one or very few types of cells. Therefore comparing different cell types side-by-side for their responses to the magnetic fields is strongly needed to achieve a better understanding for the biological effects of magnetic fields.

In comparison to Dynamic/Time-varying Magnetic Fields, static magnetic field (SMF) is more suitable to study the biological effects and their underlying mechanisms because they have less variable parameters. Electromagnetic fields from power lines, microwave ovens and cell phones are all dynamic/time-varying magnetic fields, whose effects on human bodies are still debated and causing widespread public health concerns. In contrast, SMF is characterized by steady, time-independent field strengths, and the reported biological effects of SMFs are mostly negligible or even beneficial. The core component of the MRI (magnetic resonance imaging) machines in most hospitals is a strong SMF with field intensities ranging between 0.1-3 T, in combination with pulsed radiofrequency magnetic fields. The SMF intensities in the 0.1-3 T range are currently considered to be safe to human bodies because no severe health consequences have been reported. The discomforts in patients such as dizziness are all temporary, which disappear after the MRI examination. However, mixed experimental reports from the laboratories are in the literature, which seem to be controversial. Some studies show that SMFs in this range do not affect cell growth or cell cycle [9, 10], while the others show that they may have some beneficial effects on cancer growth inhibition, either alone or in combination with chemodrugs or radiation [11–14]. Therefore, the exact effects, especially prolonged exposure of SMFs in the range of MRI machines on human bodies are still inconclusive.

Here in this study, we chose 1 T SMF to test its effect on 15 different cell lines side-by-side, including 12 human cell lines (7 solid cancer and 5 non-cancer cell lines) and 3 rodent cell lines. We found that 1 T SMF not only affected cell proliferation in a cell type-dependent manner, but also cell density-dependent manner. We revealed that cell growth of most human solid cancer cell lines we tested, but not non-cancer cell lines, can be inhibited by 1 T SMF at higher cell densities, in which the EGFR-Akt-mTOR pathway may play essential roles.

## RESULTS

### Cell type- and density-dependent cell number reduction of 1 T SMF in 7 different human cancer cell lines

**Figure 1:**
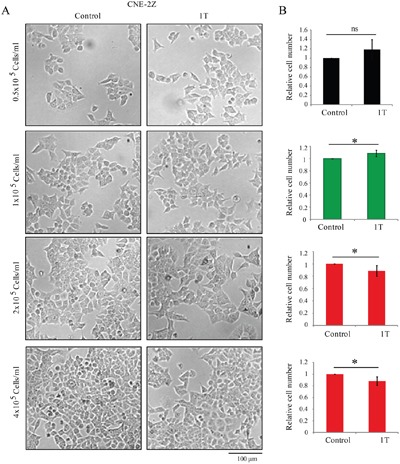
1 T Static Magnetic Field (SMF) affects the number of human nasopharyngeal carcinoma CNE-2Z cells in a cell density dependent manner CNE-2Z cells were plated one day ahead at different concentrations and treated with 1 T SMF for 2 days before they were analyzed. **A**. Representative bright field images of CNE-2Z cells after 1 T SMF exposure for 2 days. **B**. Relative cell numbers of CNE-2Z after control or 1 T SMF treatment for 2 days. Quantification was from 4 independent experiments (n=4). ns, not significant; *, p<0.05. Green color indicates 1 T SMF increases the cell number and red color indicates 1 T SMF decreases the cell number.

We previously found that 1 T static magnetic field (SMF) can effectively inhibit human nasopharyngeal cancer CNE-2Z cell proliferation [11]. However, it was interesting that we got different results when we seeded the cells at different densities. To confirm the influence of cell density on SMF-induced CNE-2Z cell proliferation inhibition, we seeded them at four different densities, 0.5, 1, 2 or 4 x 10^5^ cells/ml, cultured with or without 1 T SMF for 2 days and examined them side-by-side (Figure 1). At the end of the experiments, the control cells plated at lower cell densities are usually only around 50% confluent, while at higher cell density, the cells usually reach maximum confluence. To get unbiased and reproducible results throughout this study, we had two researchers to conduct the same sets of experiments independently and gathered their results together for statistic analysis. Experiments of four different cell densities were done using the same batch of cells side-by-side to reduce any potential variations. We found that at lower densities of 0.5-1 x 10^5^ cells/ml, 1 T SMF treatment for 2 days did not inhibit CNE-2Z cell proliferation (Figure 1). On the contrary, there was a tendency of increased cell number after SMF treatment compared to control. However, when the CNE-2Z cells were seeded at higher densities, 2-4 x 10^5^ cells/ml, it is interesting that 1 T SMF can consistently inhibit CNE-2Z cell proliferation (Figure 1). These results show that although CNE-2Z cells could proliferate in both control and 1 T SMF groups, the proliferation was differentially influenced by 1 T SMF at lower cell seeding density vs. higher cell seeding density. In another word, the cell density can directly influence the effect of 1 T SMF on CNE-2Z cells.

**Figure 2:**
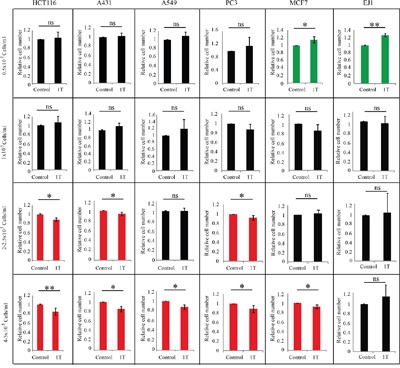
1 T SMF affects multiple human solid cancer cell lines in a cell density dependent manner HCT116, A431, A549, PC3, MCF7 and EJ1 cells were seeded at different densities one day ahead and treated with 1 T SMF for 2 days before they were counted. Relative cell numbers are shown in the figure and quantification was from 3-4 independent experiments. ns, not significant; *, p<0.05; **, p<0.01. Green color indicates 1 T SMF increases the cell number and red color indicates 1 T SMF decreases the cell number.

Next we wanted to systematically investigate multiple cell lines to find out whether cell density can also affect cells other than CNE-2Z. We chose 6 other human solid cancer cell lines, including colon cancer HCT116, skin cancer A431, lung cancer A549, breast cancer MCF7, prostate cancer PC3 and bladder cancer EJ1 cells. For each cell line, we tested four different cell densities and repeated at least three times by two independent researchers. For high densities in different cell types, we used either 4 or 5 x 10^5^ cells/ml for cell seeding, depending on which concentrations could reach maximum confluence at the end of experiments. We found that there was no significant cell morphology change by 1 T SMF treatment (Supplementary Figure 1 and 2). However, in most of them, the cell number can be reduced by 1 T SMF when they were plated at higher densities, but not at lower densities (Figure 2). In fact, SMF tends to increase cell number when they were plated at lower cell density. This indicates that cell density is an important factor that affects the impact of SMF on these human solid cancer cell lines.

### Cell type- and density-dependent effects of 1 T SMF on 5 different human non-cancer cell lines and two rodent cell lines

**Figure 3:**
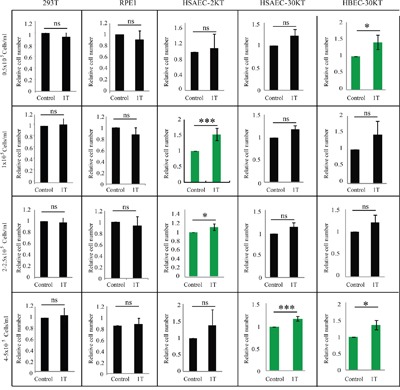
1 T SMF has minimal effects on multiple human non-cancer cell lines 293T, RPE1, HSAEC-2KT, HSAEC-30KT and HBEC-30KT cells were seeded at different cell densities one day ahead and treated with 1 T SMF for 2 days before they were counted. Relative cell numbers are shown in the figure and quantification was from 3-5 independent experiments. ns, not significant; *, p<0.05; ***, p<0.005. Green color indicates 1 T SMF increases the cell number.

The above 7 human cell lines are all human solid cancer cell lines. However, it has been shown that many non-cancer cell lines are not affected by moderate intensity SMFs. For example, Wiskirchen et al showed that 0.2, 1.0, and 1.5 T SMFs do not affect the cell growth of human fetal lung fibroblasts [9]. Next we chose 5 human non-cancer cells lines to examine the effects of 1 T SMF. We chose embryonic kidney cell line 293T, the immortalized retinal pigment epithelial cell line RPE1, and three normal lung cells (HSAEC2-KT, HSAEC30-KT and HBEC30-KT). We found that 1 T SMF did not reduce cell numbers of these human non-cancer cell lines, at all cell densities we tested (Figure 3, Supplementary Figure 3). Although the mechanism is unclear, it is obvious that the effects of 1 T SMF on these 5 human non-cancer cells lines are also cell type- and cell density-dependent (Figure 3). For 293T and RPE1 cells, 1 T SMF has minimal effects on cell numbers at all cell densities (Figure 3). For the three normal lung cells, we notice that SMF increases their cell numbers at some cell densities, which also varies between different cell types. In addition, we compared the relative cell number reduction in the 6 cancer vs. 6 non-cancer cells at four different cell densities (Supplementary Figure 4). Our results show that at higher cell density, 1 T SMF exposure for 2 days could cause ~15% cell number reduction in the 6 solid cancer cell lines, while in the 6 non-cancer cell lines there was no reduction (Supplementary Figure 4). This is statistically different (p < 0.05).

**Figure 4:**
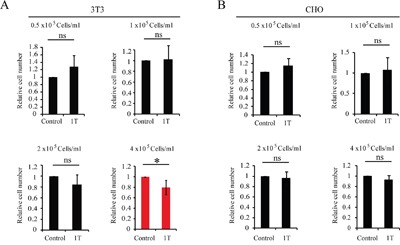
1 T SMF reduces NIH-3T3 cell number but has minimal effects on CHO cells NIH-3T3 and CHO cells were plated at different concentrations one day ahead and treated with 1 T SMF for 2 days before they were counted. Relative cell numbers are shown in the figure and quantification was from 3-5 independent experiments. ns, not significant; *, p<0.05. Red color indicates 1 T SMF decreases the cell number.

Next we examined two rodent cell lines, Chinese Hamster Ovary cell line CHO and mouse embryo fibroblast cell line NIH-3T3 (Figure 4). NIH-3T3 cells have been reported to be affected by 7-17 T SMFs [15] but CHO cells were shown to be irresponsive to SMFs ranging from moderate intensity SMFs to 13 T ultra-high SMFs in both ours and other people's studies [4, 7, 8]. Here we found that at high density, 1 T SMF reduced NIH-3T3 cell number but has minimal effects on CHO cells (Figure 4), which is consistent with previous reports. In addition, our results also show that 1 T SMF affected the NIH-3T3 cell in a cell-density dependent way. The differences between CHO and NIH-3T3 cells are probably due to the hamster vs. mouse species difference, tissue difference, or ovary vs. embryo difference, which will need more studies to disclose the underlying mechanism.

### 1 T SMF does not have obvious effects on cell death or cell cycle

**Figure 5:**
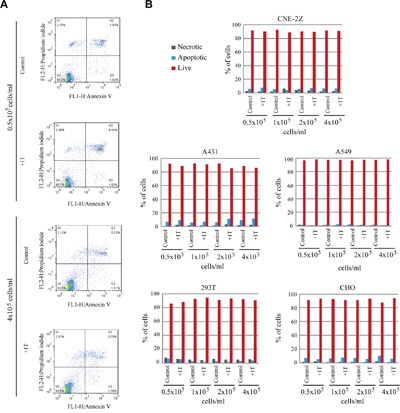
1 T SMF does not promote cell death Various cells were plated at different concentrations one day ahead and treated with 1 T SMF for 2 days before they were analyzed for cell death using Annexin/PI stain and flow cytometry. Representative raw data **A**. and quantification of live, apoptotic and necrotic cell numbers **B**. are shown.

By now we have examined 7 human solid cancer, 5 human non-cancer and two rodent cell lines, in which 1 T SMF induced differential effects on their cell numbers (Table 1). Since reduced cell number could result from reduced cell proliferation, increased cell death, or cell cycle arrest, we first examined whether the cell death was affected by 1 T SMF. We used Annexin/PI stain and flow cytometry to examine live cells, apoptotic cells or necrotic cells (Figure 5). Our results show that 1 T SMF does not have apparent effects on apoptotic or necrotic cell number, which indicates that 1 T SMF does not promote cell death in these cell lines we tested (Figure 5).

**Table 1:** A table summarizes the cell lines we used and the effects of 1 T SMF 2 day exposure on their cell numbers

	Cell line names	Species	Cell line information	effects of 1 T SMF on cell number	
				High cell density	Low cell density
**Human cancer**	CNE-2Z	human	Nasopharyngeal cancer	**Reduction**	**Increase**
	HCT116	human	colon cancer	**Reduction**	No effect
	A431	human	skin cancer	**Reduction**	No effect
	A549	human	lung cancer	**Reduction**	No effect
	MCF7	human	breast cancer	**Reduction**	**Increase**
	PC3	human	prostate cancer	**Reduction**	No effect
	EJ1	human	bladder cancer	No effect	**Increase**
**Human non-cancer**	HSAEC2-KT	human	normal lung	**Increase**	**Increase**
	HSAEC30-KT	human	normal lung	**Increase**	No effect
	HBEC30-KT	human	normal lung	**Increase**	**Increase**
	RPE1	human	retinal pigment epithelial	No effect	No effect
	293T	human	embryonic kidney	No effect	No effect
**Rodent**	CHO	hamster	Chinese Hamster Ovary	No effect	No effect
	NIH-3T3	mouse	mouse embryo fibroblast	**Reduction**	No effect

**Figure 6:**
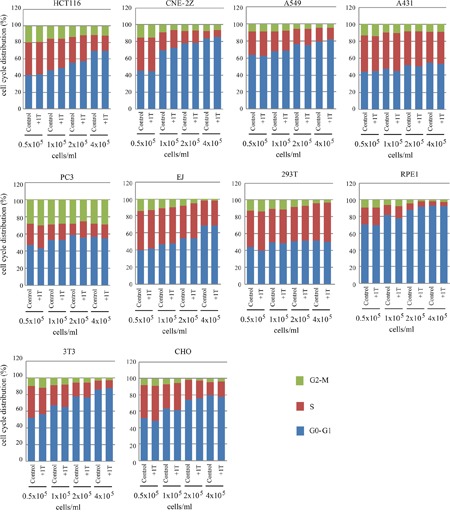
1 T SMF has minimal effects on cell cycle Various cells were plated at different concentrations one day ahead and treated with 1 T SMF for 2 days before they were analyzed for cell cycle. Experiments have been down for at least two times for each cell line and representative quantification results are shown.

Next we used flow cytometry to examine the cell cycle distribution to see whether the cell number reduction was due to cell cycle arrest at certain stage. However, although the cell density itself has a significant impact on cell cycle distribution in multiple cell lines, 1 T SMF does not have evident effect on cell cycle in all cell lines we tested (Figure 6). These results indicate that the cell number reduction by 1 T SMF we observed in multiple human solid cancer cell lines as well as the mouse embryo fibroblast NIH-3T3 cells at high density was not due to increased cell death or cell cycle arrest.

### The EGFR-Akt-mTOR pathway contributes to the differential cellular effects of 1 T SMF

**Figure 7:**
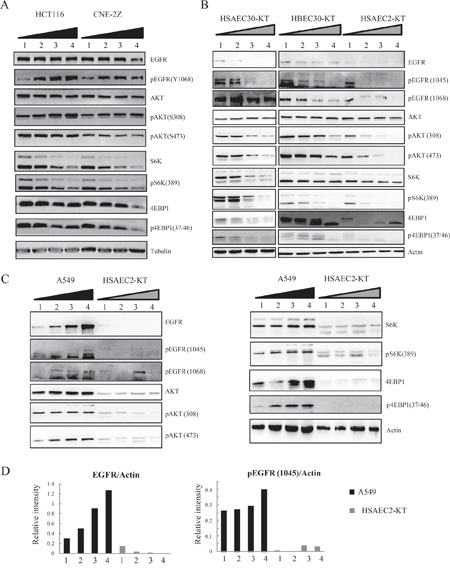
Cell density differentially affects the EGFR-Akt-mTOR pathway in cancer vs non-cancer cells **A**. Colon cancer HCT116 and nasopharyngeal cancer CNE-2Z or **B**. three normal lung cell lines HSAEC-30KT, HBEC-30KT and HSAEC-2KT were plated at four different cell densities one day ahead before they were harvested for Western Blot (WB). Representative Western Blots are shown. **C**. Lung cancer A549 and normal lung cells HSAEC-2KT cells were plated at four different cell densities one day ahead before they were harvested for Western Blot. Samples were loaded on the same gel for comparison. Representative Western Blots are shown. **D**. Quantification of relative intensity of WB in (C) to compare the difference between lung cancer vs. normal lung cells. Cropped WB images are shown in the figures to save space. Full size WB images are in Supplementary Figures 5-7.

Our results so far showed that both cell type and cell density can directly influence the effects of SMFs on cell number. However, the mechanism is still unclear. Our previous study showed that EGFR (epidermal growth factor receptor) and the mTOR pathway could be affected by SMFs [4, 11]. Specifically, using purified proteins and high resolution single molecular imaging, we previously found that SMF could directly change EGFR protein orientation and inhibit their activation. Therefore we wanted to test whether the EGFR-Akt-mTOR pathway is different at various cell densities. Western blot analysis showed that multiple components in the EGFR-Akt-mTOR pathway were clearly affected by cell density in both HCT116 and CNE-2Z cancer cell lines (Figures 7A and Supplementary Figure 5). For example, the phosphorylation level of EGFR and Akt were increased when the cells were plated at higher density. It is interesting that the EGFR-Akt-mTOR pathway was also affected in three normal human lung cell lines by cell density (Figure 7B and Supplementary Figure 6) but the change pattern was different. More specifically, the phosphorylation level of EGFR and Akt were decreased at higher cell density in these normal lung cells, which are opposite to HCT116 and CNE-2Z cancer cells.

Next we compared the cancer vs. non-cancer cells from human lungs. We examined different densities of A549 lung cancer cells and HSAEC2-KT normal lung cells side-by-side to see whether the EGFR-Akt-mTOR pathway is differentially regulated by cell type and cell density (Figure 7C and Supplementary Figure 7). Not surprisingly, multiple components in the EGFR-Akt-mTOR pathway were highly expressed/activated in the A549 lung cancer cells but not in HSAEC-2KT normal lung cells (Figure 7C, 7D and Supplementary Figure 7), which is consistent with their oncogenetic functions. In addition, the cell density also affected their expression differentially in these two cell lines. Although the protein expression and phosphorylation pattern in the three cancer cell lines we tested here (HCT116, CNE-2Z and A549) are not completely identical, they have some aspects in common. For example, increasing cell density in HCT116, CNE-2Z and A549 lung cancer cells increased the expression and phosphorylation of EGFR, but not in HSAEC2-KT normal lung cells. Since EGFR is an important anti-cancer target that is overexpressed in multiple cancer cells [16–19] and is also a direct target for SMFs [4], we hypothesized that it may played a key function in SMF-induced cell type and density-dependent cell proliferation inhibition.

**Figure 8:**
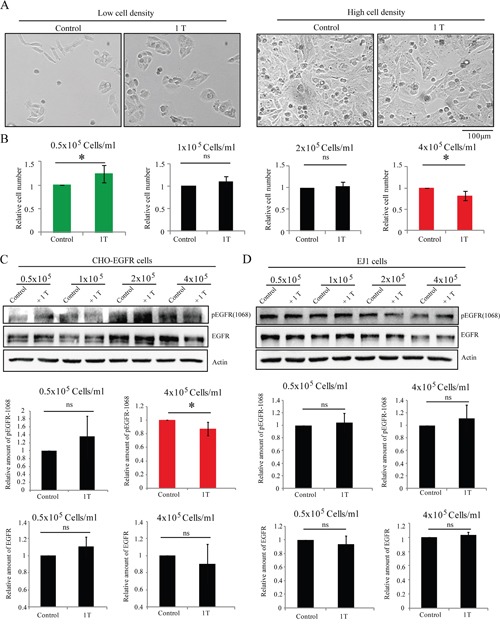
The EGFR expression influences SMF-induced cell proliferation effects CHO cells stably transfected with EGFR-FLAG (CHO-EGFR cells) or EJ1 cells were plated at different cell densities one day ahead, and treated with 1 T SMF for 2 days before they were counted for cell number **A, B**. and analyzed for Western Blot **C, D**. (A) Representative bright field images of cells. (B) Quantification of (A) from 4 independent experiments. ns, not significant; *, p<0.05. Green color indicates 1 T SMF increases the cell number and red color indicates 1 T SMF decreases the cell number. (C, D) Representative Western Blots and the quantification results. EGFR and pEGFR were normalized to actin or tubulin control.

Next we tested whether EGFR can convert the EGFR-null CHO cells (Figure 4) from SMF-insensitive into SMF-sensitive cells by EGFR transfection. We made a CHO cell line that stably overexpresses EGFR with a FLAG tag (CHO-EGFR cells) [4]. Here we examined them for their responses to 1 T SMF at different cell densities. Our results showed that its cell number was reduced by 1 T SMF in a cell density-dependent manner (Figure 8A), which was different from CHO cells (Figure 4), but was similar to many cancer cells we tested (Figure 2). Quantification results showed that at 0.5 x 10^5^ cells/ml, the cell number of CHO-EGFR cells were increased by 1 T SMF (Figure 8B). However, when they were plated at 8-fold higher density (at 4 x 10^5^ cells/ml), the cell number of CHO-EGFR cells are reduced by 1 T SMF (Figure 8B). These results show that transforming EGFR, a protein that is overexpressed and/or activated in multiple cancers, can make the SMF-insensitive CHO cells respond to SMF in a cell density-dependent manner. In addition, Western blot analysis also showed that the EGFR expression level and phosphorylation at 1068 were both increased in cells plated at higher cell densities, which were similar to HCT116, CNE-2Z and A549 cancer cells (Figure 8C). In addition, the EGFR phosphorylation level at higher cell density could be reduced by SMF (Figure 8C). In contrast, the EJ1 cell, which was the only cancer cell line in all 7 solid cancer cell lines we tested that was not reduced by 1 T SMF, showed a different EGFR pattern. The EGFR expression level and phosphorylation level do not increase at higher cell density (Figure 8D). Moreover, the EGFR phosphorylation level of EJ1 cells at higher cell density could not be reduced by 1 T SMF (Figure 8D). Therefore, although we cannot exclude the possible involvement of other cellular factors, for example, other Receptor Tyrosine Kinases that are also overexpressed in many cancer cells, our data show that the EGFR-Akt-mTOR pathway is one of the key factors involved in the SMF-induced differential effects in different cell types at different densities. EGFR is likely to be at least one of the major reasons that contribute to the 1 T SMF-induced cell number reductions in some solid cancer cell lines.

### 1 T SMF increases the efficacy of Akt inhibitors on CNE-2Z cell growth inhibition

**Figure 9:**
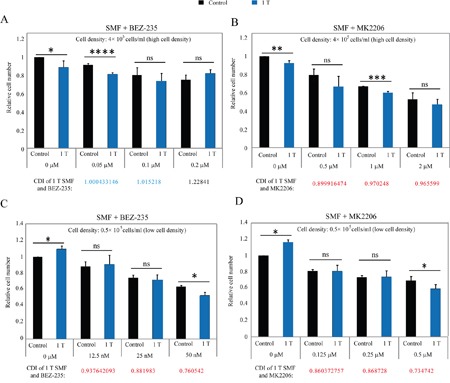
1 T SMF increases the efficacy of AKT inhibitors on CNE-2Z cell inhibition CNE-2Z cells were plated one day ahead at 4 x 10^5^ or 0.5 x 10^5^ cells/ml and treated with 1 T SMF with or without different BEZ-235 or MK2206 for 2 days before they were counted. **A, B**. High cell density (4 x 10^5^ cells/ml). **C, D**. Low cell density (0.5 x 10^5^ cells/ml). Relative cell numbers are shown and quantifications were from 3-4 independent experiments by two independent researchers. CDI (Coefficient of drug interaction) values are shown. Blue color indicates additive effect. Red color indicates weak synergistic effect. ns, not significant; *, p<0.05; **, p<0.01; ***, p<0.005; ****, p<0.001.

We have previously shown that 1 T SMF can increase the drug efficacy of EGFR and mTOR inhibitors [4, 11]. Since EGFR and Akt inhibitors are frequently used in combinational therapy to improve the drug efficacy [20, 21], next we examined whether 1 T SMF can also increase the drug efficacy of Akt inhibitors (BEZ-235 and MK2206) (Figure 9). We tested them at both high cell density (Figure 9A, 9B) and low cell density (Figure 9C, 9D). We found that although the combinational effects were more consistent and obvious in some drug concentrations than the others, overall the drug efficacies of both Akt inhibitors (BEZ-235 and MK2206) were increased by 1 T SMF (Figure 9). To quantitatively analyze the combination effects between 1 T SMF and Akt inhibitors, we calculated their coefficient of drug interaction (CDI) (Figure 9). Most CDI values are between 0.7 and 1, which indicate there are weak synergistic effects between SMF and these two Akt inhibitors on cell number reduction in CNE-2Z cancer cells.

## DISCUSSION

We suspected that the cell density-induced variations must have contributed to some of the inconsistencies in the literature. Most researchers in the field of biological studies of magnetic fields, actually including us, did not pay much attention to the cell density, or at least did not realize that the cell density could cause dramatic differences in our experimental outcomes. However, it has been shown that the cell density difference could directly cause variations in cell growth rate, protein expression, as well as alterations in some signal pathways [22–28]. Therefore, although our data in this study did not provide clear-cut molecular mechanisms for the SMF-induced cellular effects in all types of cells at all cell densities, we aim to alert people that both cell type and density are key factors that directly influence the effects of SMF on cell proliferation. Researchers should take these into account when they analyze their own data as well as the current literature.

The apparent different responses of human cancer vs. non-cancer cells to 1 T SMF are very interesting. Actually there were multiple previous studies indicated that SMFs have more impacts on cancer cells than on non-cancer cells [5, 14, 29] and moderate intensity SMFs could increase the efficacy of some chemodrugs on cancer cells [11, 12, 30, 31]. Moreover, moderate intensity SMF was also shown to be able to reduce tumor growth and increase chemodrugs efficacy in mouse models [13, 30]. Here we found that the inhibition effects of 1 T SMF on cancer cells are cell type- and cell density- dependent. We showed that for 6 out of 7 human solid cancer cell lines, 1 T SMF reduced their cell numbers at higher cell densities but not at lower cell densities. In contrast, for all 5 human non-cancer cell lines, 1 T SMF did not reduce cell numbers at all cell densities we tested. For the same tissue, the cell number of human lung cancer A549 cells was effectively reduced by 1 T SMF at higher cell density, but cell number of normal human lung cells HSAEC2-KT, HSAEC30-KT and HBEC30-KT were even increased.

The fact that 1 T SMF could inhibit many solid cancer cells growth at higher densities is interesting and potentially promising. Although the regular MRI examination in the hospitals only last minutes to hours, the SMF could be provided by other devices. There are actually some people using permanent magnets to treat late-stage cancer patients/volunteers and got encouraging results (unpublished). Their treatment time goes far beyond the MRI machines. However, our results showed that the cell growth for one of the seven cancer cell lines, the bladder cancer EJ1 cell, was not reduced by SMF at all. In addition, we have only tested 7 human solid cancer cell lines, whether other cancer cells in suspension, such as various types of leukemia cells, can be affected in the same pattern is still unknown. Therefore, although at higher cell density, 1 T SMF reduced the cell number in majority of cancer cell lines we tested, further investigations are strongly needed to explore the clinical potential of SMFs on cancer growth inhibition. It is very possible that only some cancer cell types can be inhibited by SMFs, which is likely relevant to their different genetic background. In addition, the tendency of SMFs to promote some cancer cell growth at lower cell density should also raise caution about the potential oncogenic effect at early cancer stages for this prolonged SMF exposure. More investigations are needed to address this issue.

One limitation of our study was that we did not address the potential issue of metabolism, nutritional competition for cells at high density, which is apparently more stringent than cells at lower density. Theoretically, SMFs may affect cell metabolism, for example, utilization of carbon and nitrogen sources, oxygen consumption, respiration etc. However, we recently started to examine two types of leukemia cells in suspension and found that 1 T SMF inhibited both of them at lower cell concentration but not at higher cell concentrations. Therefore we think it is less likely that the reasons mentioned above such as oxygen consumption or nutrient competition are the major factors. In the meantime, it has been shown that moderate intensity SMFs could affect intracellular signaling pathways [32, 33]. Although we found that the EGFR-Akt-mTOR pathway is involved, other factors must co-exist. Further investigations are strongly needed to examine more cell types at different conditions to unravel additional mechanisms of the SMF effects on various cancer cell types at different conditions.

It was known that the biological effects of magnetic fields can be influenced by the magnetic field types, strength, frequency, treatment time and other parameters, which all contribute to the mixed results of biological effects of magnetic field in the literature. Here by systematically testing 15 different cell lines at 4 different cell densities, we revealed that both cell types and densities are key factors that influence the effects of SMF. In addition, EGFR-Akt-mTOR likely contributes to the cell types and densities induced variations. The fact that most cancer cell lines can be inhibited at high density indicates the clinical potential of SMF in solid tumors or end-stage tumors.

## MATERIALS AND METHODS

### Cell culture

We tested 15 cell lines in total, which are all adherent cell lines. For the 12 human cell lines, we chose 7 cancer cell lines (nasopharyngeal cancer CNE-2Z, colon cancer HCT116, skin cancer A431, lung cancer A549, breast cancer MCF7, prostate cancer PC3 and bladder cancer EJ1 cells) and 5 non-cancer cell lines (embryonic kidney cell line 293T, immortalized retinal pigment epithelial cell line RPE1, and three normal lung cell lines HSAEC2-KT, HSAEC30-KT and HBEC30-KT). The three rodent cell lines are Chinese Hamster Ovary cell line CHO and mouse embryo fibroblast cell line NIH-3T3, as well as CHO cells that overexpresses EGFR-FLAG (CHO-EGFR cells). All cells were from ATCC except for CHO-EGFR, which was constructed as previously described [4]. HCT116, A431, A549, MCF7, PC3, 293T, NIH-3T3, CHO, CHO-EGFR and RPE1 cells were maintained in DMEM (Dulbecco minimum essential medium) supplemented with 10% (vol/vol) fetal bovine serum (FBS) and 1% (vol/vol) penicillin and streptomycin (P/S). CNE-2Z and EJ1 cells were cultured in RPMI-1640 supplemented with 10% FBS and 1% P/S. HSAEC2-KT, HSAEC30-KT and HBEC30-KT cells were cultured in SAGM medium. All cells were cultured in 5% CO2 at 37 °C.

### Magnetic field exposure

Cells were plated at different concentrations one night ahead to allow them to attach to the tissue culture plates. Cells plated at 1 x 10^5^ cells/ml are equivalent to the density of 2 x 10^4^ cells/cm^2^. On the second day, they were placed in regular full-sized CO_2_ cell incubator (Shanghai Boxun, BC-J160S) that has accurate control of temperature (37°C), humidity and CO_2_ (5%) (Supplementary Figure 8A, 8B). The sham control group (control) was placed far away from the magnets (dimension: 5 cm x 5 cm x 5 cm) and the Gauss meter (LakeShore 475 DSP Gaussmeter) showed the magnetic field intensity of 0.925 ± 0.206 Gs (background magnetic field in the lab was 0.875 ± 0.171 Gs and in a separate CO2 cell incubator with no magnets was 0.875 ± 0.096 Gs). The sham control is labeled as “control” in the figures. The magnetic exposure group (+ 1 T) was placed directly on the top center of the magnets, where the Gauss meter showed that the magnetic intensity was 1.07 ± 0.037 T. The magnetic field exposed cells were labeled as “ + 1 T” in the figures. The magnetic field exposure group (1.07 ± 0.037 T) has around 10,000-fold higher magnetic field intensity than the sham control (0.925 ± 0.206 Gs).

The magnet surface dimension is 5 cm x 5 cm and the diameter of the cell culture plates we used was 3.5 cm (Supplementary Figure 8C). Therefore the whole cell culture plate was fully covered by the magnet. Both control and the 1 T SMF exposed plates were in the same incubator to reduce experimental variations. For cells treated with SMF and Akt inhibitors, the inhibitors were added right before the cells exposed to sham control or the 1 T SMF. Cells were incubated with or without 1 T SMF for 2 days before they were taken out and subjected to further analyses, including cell counting, cell cycle and cell death analysis and Western blotting.

### Cell counting

As mentioned above, cells were plated at different concentrations in 35 mm cell culture plates. After SMF exposure, bright field images were taken before the cells were harvested by trypsinization. An aliquot of the cells were counted by hemocytometer and the rest cells were used for flow cytometry analysis (cell death and cell cycle). Experiments were repeated for at least three independent times by two researchers. The results were gathered together for analysis.

### Cell cycle analysis

Cells were trypsinized and washed with PBS. Then they were fixed in 70% ice-cold ethanol overnight at 4 °C before they were washed with PBS, and incubated in PI (propidium iodide) solution (BD Pharmingen) for 30 min at room temperature in the dark. Samples were then analyzed on a BD Flow Cytometry (BD Biosciences, Calibur). For each condition, we collected 1 x 10^4^ cells per sample. Data were analyzed using ModFit LT. Experiments were done for at least two times and representative results were shown in the figures.

### Annexin V/PI double stain

Cells were trypsinized and washed twice with ice-cold PBS before they were resuspended in binding buffer at 10^6^ cells/ml. Then 100 μl of them was transferred to a 5 ml culture tube and 5 μl of FITC-Annexin V + 5 μl of PI (FITC-Annexin V Apoptosis Detection Kit was from BD Pharmingen^TM^) were added to the tube, mixed, and incubated in the dark for 15 min at room temperature. Then, 400 μl of binding buffer was added to the stained cells before they were analyzed by flow cytometry within 1 h. Approximately 1 x 10^4^ cells were collected by flow cytometer. Experiments were done for at least two times and representative results were shown in the figures.

### Western blotting

Cells grown in tissue culture plates were lysed directly in plates by 200 or 100 μl of M-PER lysis buffer (Pierce) supplemented with protease and phosphatase inhibitor cocktail at 4°C for 20 min. The whole cell lysate was mixed with 2 x SDS loading buffer, boiled, and subjected to Western blotting. The PVDF membrane was blocked with 5 % NFDM (non-fat dry milk) at room temperature for around 1 h. Corresponding primary antibodies were diluted in AbDil-Tween (TBS; 2 % BSA; 0.1 % Tween-20) at 1:1000 dilution. Primary antibodies used include phospho-specific antibodies, EGFR, Akt, S6K, 4EBP1, and the HRP-linked anti-rabbit and anti-mouse IgG antibodies which were from Cell Signaling Technology. The mouse monoclonal antibodies for beta-tubulin and beta-actin were from Beijing TransGen Biotech. All primary antibodies were diluted in AbDil-Tween at 1:1000 dilution (TBS supplemented with 2% BSA and 0.1% Tween-20) and HRP conjugated secondary antibodies were diluted in TBS with 0.1 % Tween-20 and 5 % NFDM at 1:5000 dilution. Western blotting results were obtained by Bio-Rad ChemiDoc^TM^ XRS+ System and Beijing Tanon Fine-do X6. ImageJ software was used to quantify the protein relative level shown by Western blots.

### Statistical analysis

To ensure the reproducibility of the experiments, most experiments in this work were repeated by two independent researchers on different days. Each researcher did their experiment independently, according to the same protocol. For quantifications presented in the figures, experiments were repeated for at least three independent times (n=3). In each experiment, one plate of sham control and one plate of magnetic exposure were examined. Each plate had 1-10 x 10^5^ cells seeded at the beginning of the experiment.

For quantifications in this manuscript, mean values are shown in all figures, and standard deviations are shown as error bars. All images shown in figures are representative results from multiple experiments. Comparisons between different treatments were analyzed by a two-tailed Student t test. P values are labeled in figures for where data were compared.

We calculated the coefficient of drug interaction (CDI = AB/(A x B)) of SMF in combination with Akt inhibitors for their effects on cell numbers in Figure 9. AB is the ratio of SMF + drug combination group to the control group. A or B is the ratio of SMF or drug single treatment group to the control group. Generally a CDI value < 1 indicates synergistic effect, a CDI value = 1 indicates additive effect, a CDI value > 1 indicates antagonistic effect. Usually CDI < 0.7 indicates that the treatment combination is significantly synergistic.

## SUPPLEMENTARY MATERIALS FIGURES


