# Supplementary material for: Cell type- and density-dependent effect of 1 T static magnetic field on cell proliferation
Source: Oncotarget. 2017 Jan 4;8(8):13126–41. doi: 10.18632/oncotarget.14480 (PMC5355082; doi:10.18632/oncotarget.14480)
Supplement: Supplementary file 1 [file oncotarget-08-13126-s001.pdf]

Cell type- and density-dependent effect of 1 T static magnetic field on cell proliferation

SUPPLEMENTARY FIGURES

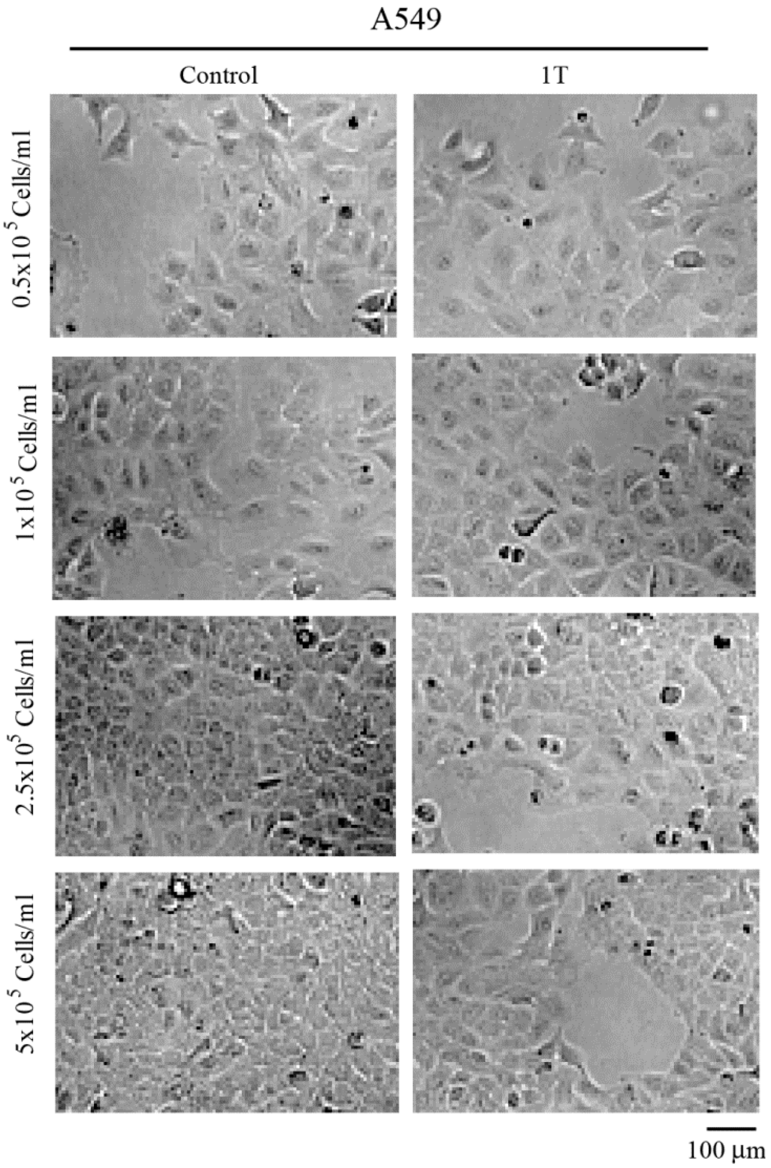

Supplementary Figure 1: 1 T Static Magnetic Field reduces the number of human lung cancer A549 cells seeded at higher cell density but not at lower cell density. A549 cells were seeded one day ahead at different concentrations and treated with 1 T SMF for 2 days. Representative bright field images are shown.

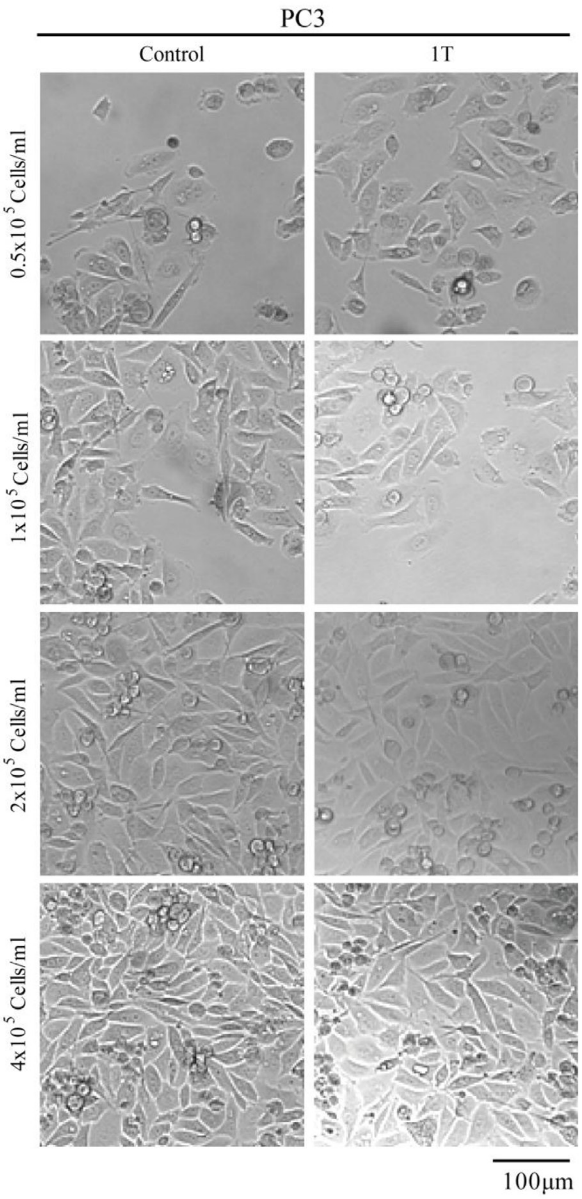

Supplementary Figure 2: 1 T Static Magnetic Field reduces the number of human prostate cancer PC3 cells at higher density but not at lower cell density. PC3 cells were plated one day ahead at different concentrations and treated with 1 T SMF for 2 days. Representative bright field images are shown.

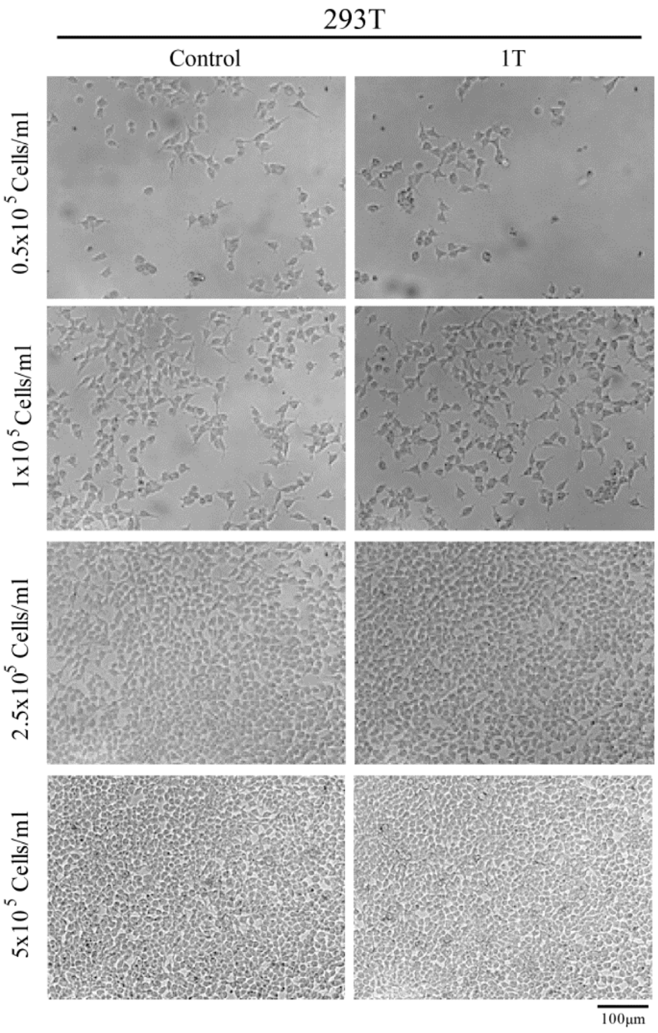

Supplementary Figure 3: 1 T Static Magnetic Field does not affect the number of human embryonic kidney 293T cells. 293T cells were plated one day ahead at different concentrations and treated with 1 T SMF for 2 days. Representative bright field images are shown.

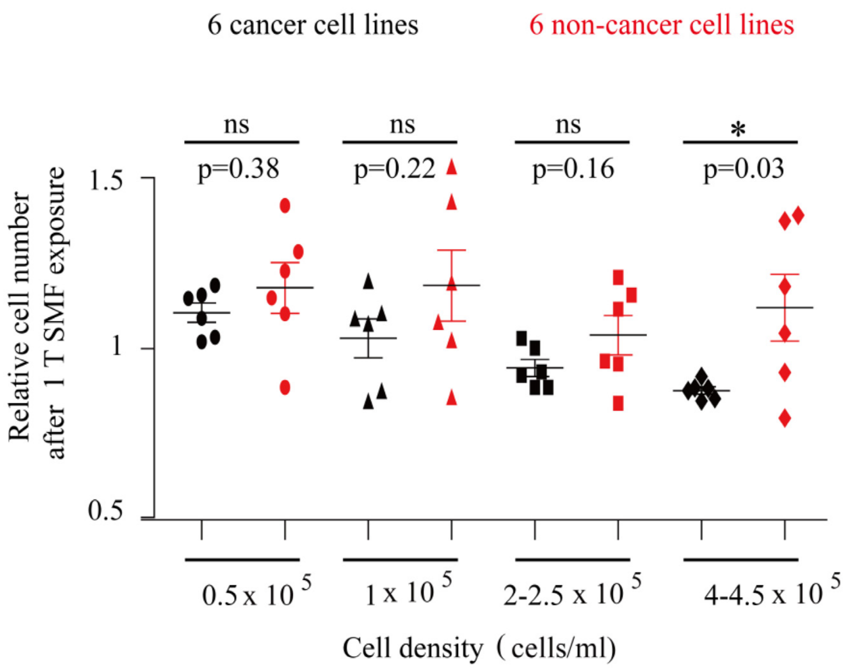

Supplementary Figure 4: The differential effects of 1 T SMF on cell numbers in 6 solid human cancer cell lines and 6 non-cancer cells lines. The 6 human solid cancer cell lines (show in black) include human nasopharyngeal cancer CNE-2Z, colon cancer HCT116, skin cancer A431, lung cancer A549, breast cancer MCF7 and prostate cancer PC3. The 6 non-cancer cell lines (show in red) include embryonic kidney cell line 293T, immortalized retinal pigment epithelial cell line RPE1, and three normal lung cell lines HSAEC2-KT, HSAEC30-KT and HBEC30-KT, and Chinese Hamster Ovary cell line CHO. Each dot represents the mean value of relative cell number of 1 T SMF treated cells relative to control condition calculated from Figures 1-4. P values are labeled in the figure. “ns”, not significant; “*”, $p < 0.05$.

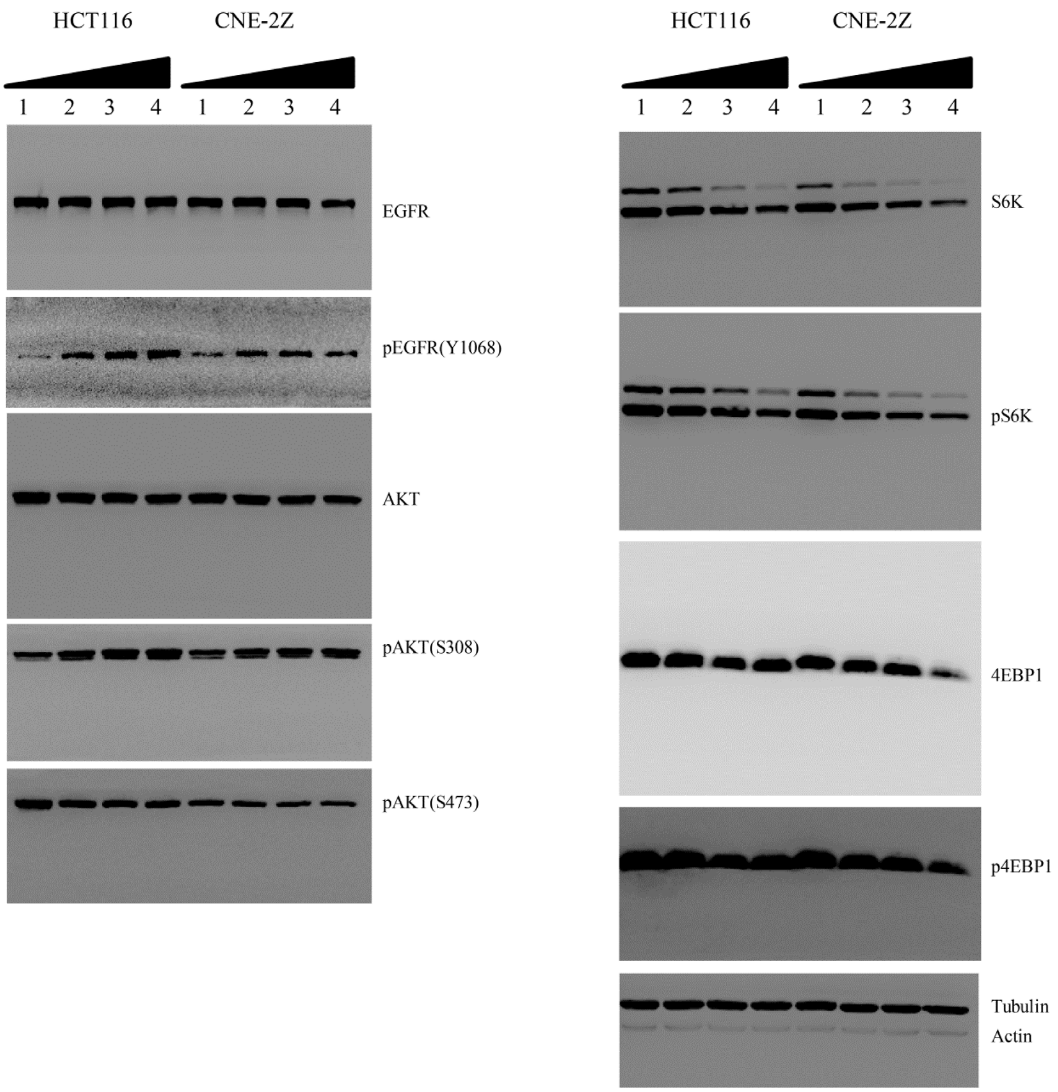

Supplementary Figure 5: Cell density affects the EGFR-Akt-mTOR pathway in HCT116 and CNE-2Z cancer cells. These are the full-size Western blot images of Figure 7A.

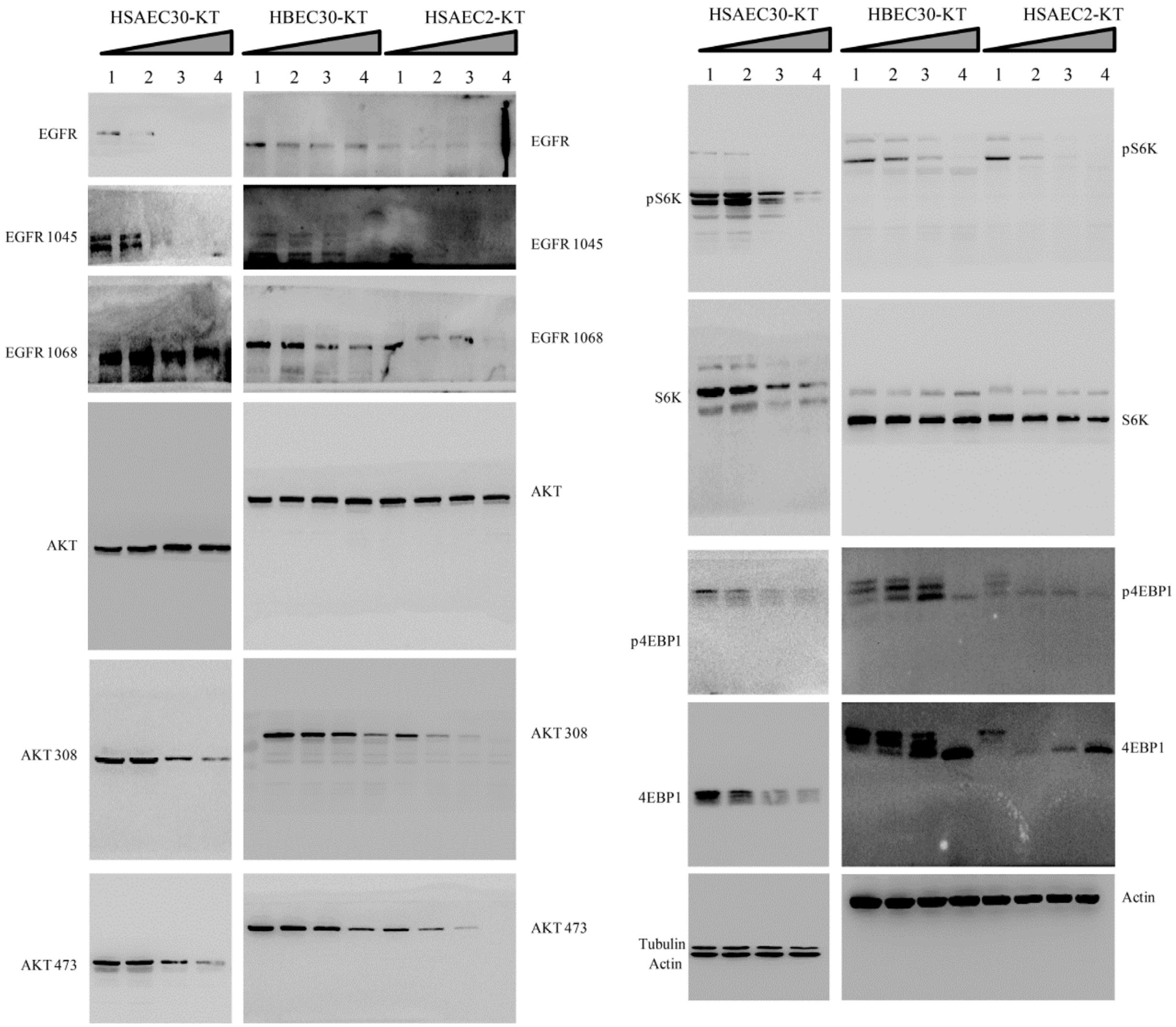

Supplementary Figure 6: Cell density affects the EGFR-Akt-mTOR pathway in three normal lung cell lines. These are the full-size Western blot images of Figure 7B.

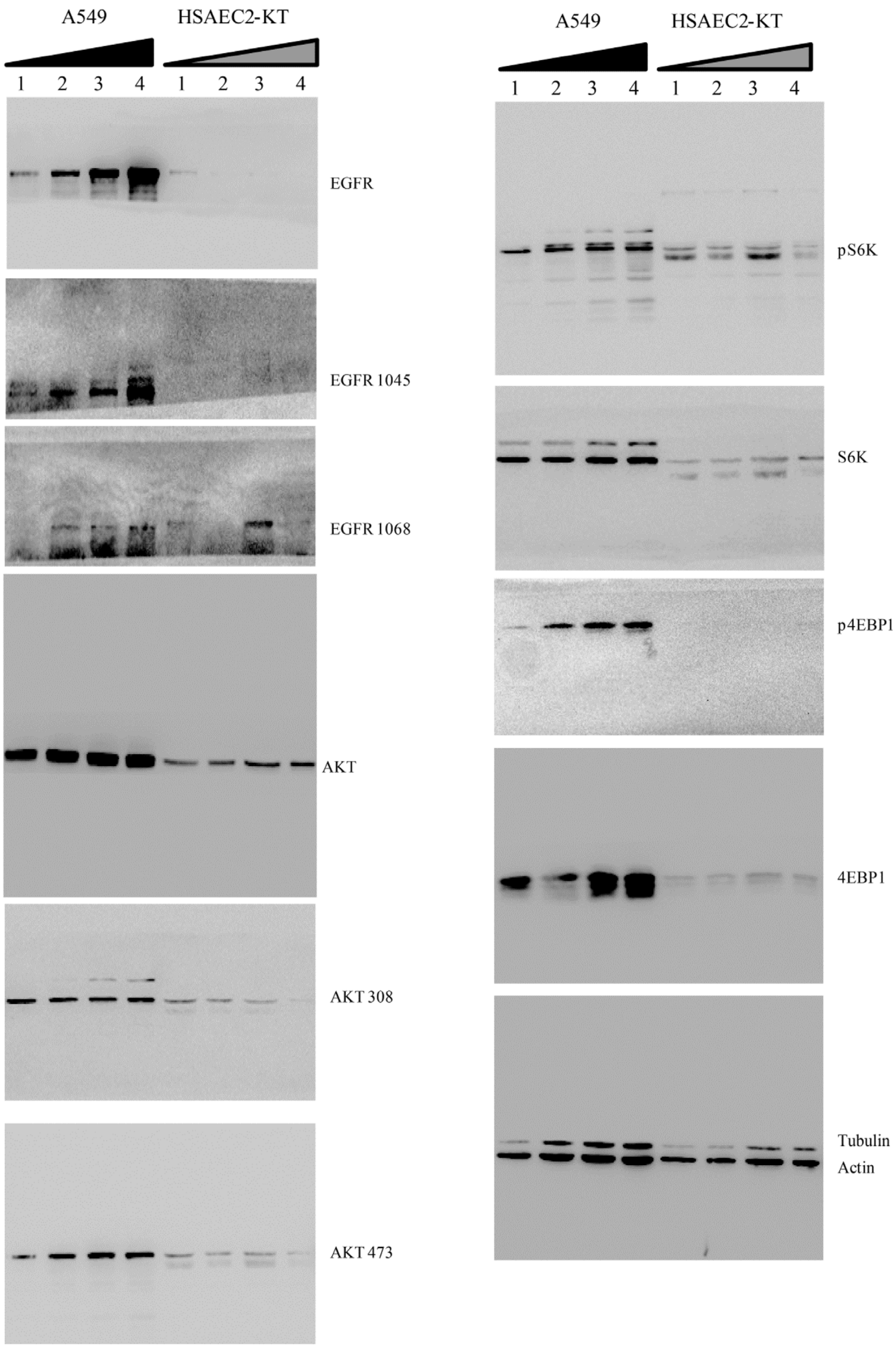

Supplementary Figure 7: EGFR-Akt-mTOR pathway is regulated by both cell type and cell density in lung cancer A549 vs. normal lung cells HSAEC2-KT cells. These are the full-size Western blot images of Figure 7C.

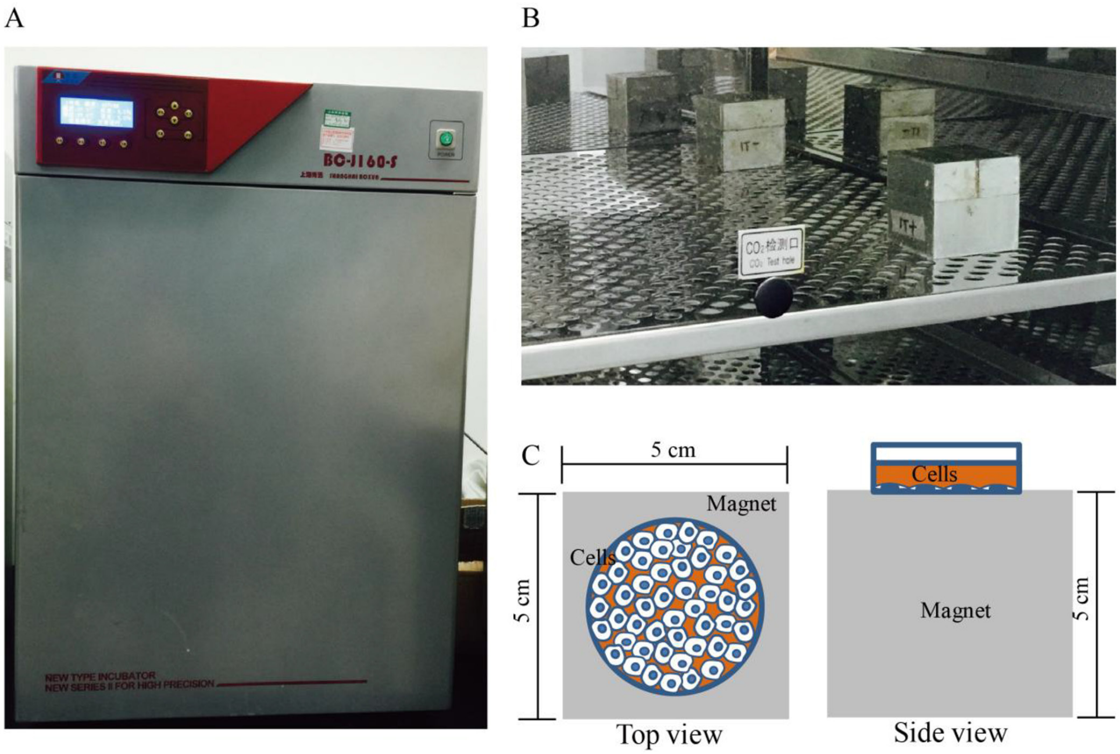

Supplementary Figure 8: The CO_2 Cell incubator and magnetic field exposure. A. The full size CO_2 cell incubator. Dimension: 48cm (L), 47 cm (W), 60cm (H). **B.** Magnets placed on one shelf within the incubator. **C.** Illustration of the cell culture plate and the magnet. Magnet dimension: 5 cm (L), 5 cm (W), 5 cm (H). The diameter of the cell culture plate is 3.5 cm.
